# Ultra-Performance Liquid Chromatography-Quadrupole-Time-of-Flight-Mass Spectrometry-Based Analysis of Facial Physiological Parameters and Lipid Composition of Between Sensitive Skin of Women Aged 36–42 and 43–49 Year

**DOI:** 10.3390/life15020175

**Published:** 2025-01-25

**Authors:** Yu Li, Rong Tang, Lizhi Yue, Congfen He

**Affiliations:** Key Laboratory of Cosmetics, China National Light Industry, College of Light industry Science and Engineering, Beijing Technology and Business University, Beijing 100048, China; 15376561326@163.com (Y.L.); yuelizhi11@163.com (L.Y.)

**Keywords:** sensitive skin, age, lipid, correlation analysis, skin aging

## Abstract

Background: UPLC-Q-TOF-MS (Ultra-Performance Liquid Chromatography–Quadrupole Time-of-Flight Mass Spectrometry) is a high-precision, high-density technology for lipid analysis. Sensitive skin is a sub-stable condition, and it has been reported that the population of sensitive skin in China is predominantly female. Meanwhile, women with sensitive skin have different physiological parameters as well as lipid compositions at different ages. The Yellow Emperor’s Classic of Internal Medicine states that the number of women’s life cycles is seven, that major changes are manifested every 7 years, and that aging begins at age 35. At present, the correlation between facial lipid composition and aging indicators of sensitive skin in women aged 36–42 and 43–49 years has not been reported. Objective: This study reveals the relationship between key lipid composition of the facial skin and the aging of sensitive skin in women aged 36–42 and 43–49 years. Methods: We used UPLC-Q-TOF-MS technology to study the changes in lipid composition in the sensitive skin of woman aged 36–42 and 43–49 years, using a multi-probe adapter system with different types of skin-testing probes to test physiological parameters. Three types of multivariate data—questionnaires, physiological indicators, and lipid composition—were used together to assess differences in aging in a population of women with sensitive skin at different ages. Results: 1. In the questionnaire part, the T1 group was more susceptible to sunburn and the T2 group was more susceptible to tanning. 2. In the physiological index part, the aging characteristics of facial skin in the T2 group were obvious, with the b-value, as well as the brown area size, being significantly higher than the T1 group, while the TWEL, sebum, R2 value, ITA value, pore count, and concentration of the red area were significantly lower than the T1 group. 3. In the lipid part, the total facial lipid content was higher in the T2 group, with a significantly higher GP lipid, and the 47 VIP lipids obtained were analyzed by ROC curves, narrowing down to six lipids, PS(2-OMe-21:0/0:0), PS(O-18:0/20:5 (5Z,8Z,11Z,14Z,17Z)), PA(O-16:0/20:5 (5Z,8Z,11Z,14Z,17Z)), PS (P-16:0/12:0, PA (O-16:0/22:2 (13Z,16Z)), and PC (19:3 (10Z,13Z,16Z)/0:0)), and all six lipids were higher in the T2 group. 4. In Spearman correlation analysis, PS(O-18:0/20:5(5Z,8Z,11Z,14Z,17Z)), PS(P-16:0/12:0), PS(2-OMe-21:0/0:0), PA(O-16:0/20:5(5Z,8Z,11Z,14Z,17Z)), and PC(19:3( 10Z,13Z,16Z)/0:0), which are five lipids and skin aging indicators (TWEL, sebum, ITA value, b-value, pore count, concentration of red area, and brown area size) were significantly correlated. Conclusions: Through correlation analysis, it was found that changes in the composition of skin surface lipids (SSLs) in both age groups have an important influence on facial physiological indicators (aging manifestations) and played an important role in furthering the understanding of sensitive skin aging. Therefore, these lipid components also provide theoretical support for the development of cosmetic ingredients that slow down the aging of sensitive skin.

## 1. Introduction

Sensitive skin (SS) is a well-known skin condition showing sensory irritation to daily used products such as cosmetics or pharmaceuticals, possibly containing sensory irritants [1]. Epidemiological studies show that China’s sensitive skin population is about 40.8%, and women are the most common people with sensitive skin [2,3]. Aging is an inevitable physiology process, which is more prominent in humans with sensitive skin, and both genetic mechanisms and environmental factors can account for it. Physiological index parameters, including skin hydration, transepidermal water loss (TWEL), sebum content, and other indices, as well as skin surface lipid (SSL) composition, were different across different ages [4]. The classic Chinese medical text “Huangdi Neijing” puts forward the “seven–seven theory”, which believes that a woman takes “seven” as the law, in which it is believed that a woman’s physiological condition will undergo a significant change every 7 years [5]. At the age of 35, the facial skin begins to age; at the age of 42, the facial skin continues to age, and the hair begins to fall out; and at the age of 49, blood declines, menstruation stops, and the appearance continues to age. Current advances in sensitive skin research have focused on changes in physiological characteristics and SSLs between sensitive skin and non-sensitive skin at the same age. Wan Zongyuan et al. [6] recruited 40 subjects aged 25–35 years and divided them into sensitive and non-sensitive groups through a lactic acid tingling test, which found that triglyceride levels were significantly lower in the sensitive group; Ma Yuchen et al. [7] recruited 58 subjects aged 18–25 years, divided them into sensitive and non-sensitive groups, and found that Cer/GlcCer could promote lipid synthesis and secretion by upregulating lipid-related gene expression to repair barrier damage. However, the study of how sensitive skin surface lipids (SSLs) change in women and how they relate to indicators associated with skin aging in different age groups is of great significance.

UPLC-Q-TOF-MS (Ultra-Performance Liquid Chromatography–Quadrupole Time-of-Flight Mass Spectrometry) technology analyzes SSLs and qualitatively analyzes lipid compositions for untargeted lipidomics analysis. Untargeted lipidomics involves the identification and quantification of hundreds of lipids through a high-throughput process, maximizing the response to the total metabolite features of the samples to screen for lipid biomarkers [8,9]. This study used UPLC-Q-TOF-MS technology to comprehensively analyze the major classes of lipids, subclasses of lipids, and the key lipid components of sensitive skin in 36–42- and 43–49-year-old females. The skin’s physiological parameters were quantified using non-invasive instruments. Spearman’s analysis of the key lipids and physiological parameters was performed to explore the key lipid biomarkers in the aging process of sensitive skin of women of different ages, which can provide a theoretical basis for the research and development of age-specific lipocosmetic products for women with sensitive skin.

## 2. Materials and Methods

### 2.1. Reagents and Instruments

LC-MS-grade isopropanol, acetone, chloroform, ammonium formate, acetonitrile, formic acid, methanol, Beijing Bairdi, China; Sebutape^®^ sebum sampling paper, Cuderm Corporation, TX, USA; distilled water, Watson’s Hong Kong, China; dry ice, Changsha Chenghui Dry Ice Co., Ltd., Changsha, China.

ACQUITY UPLC I Class/Xevo G2-XS Q-TOF, chromatographic column CSH C18 (2.1 mm × 100 mm, 1.7 μm), Waters, Milford, MA, USA; Nitrogen Blowout Instrument, Japan; Vortex Mixer (VORTEX-5), Kylin-Bell Lab Instrument Co., Ltd., Haimen, China; Centrifuge. Eppendorf, Germany; Multi Probe Adapter System, CK, Germany; CBS-2028 cloud microscope, Wuhan Boshi Electronics Co., Ltd., Wuhan, China. High-Purity Nitrogen, Shuangquan Tianyuan Gas Co., Ltd., Beijing, China.

### 2.2. Method

#### 2.2.1. Subject

Female subjects aged 36–49 years were recruited in Beijing for the lactic acid sting test, where 50 μL of 50% lactic acid was applied to the nasolabial folds and the left cheek. Subjects were divided into two groups and asked about their autonomic status at 2.5 and 5 min, with responses scored on a four-point scale, with a total score of ≥3 on both occasions being considered positive for lactic acid tingling, qualifying the subjects for enrollment. Enrolled subjects were divided into the T1 group (36–42 years old, 33) and T2 group (43–49 years old, 34). This study is in accordance with the Declaration of Helsinki, lot number ERGZ20230517-05.

Exclusion criteria: (1) patients with a history of other skin diseases or have used hormone drugs in the last three months; (2) suffering from serious heart, liver, or kidney function impairment; (3) suffering from neurological, psychiatric, or serious endocrine diseases; (4) known to have severe immunocompromise or long-term use of corticosteroids and immunosuppressants; (5) pregnant or lactating women; (6) oral contraceptive pills taken in the last 4 weeks; (7) a tendency toward skin cancer; (8) have received systemic or topical medical aesthetic program treatment in the last 2 weeks; (9) have participated in other Chinese and Western drug clinical trials in the last 4 weeks; (10) a history of alcohol or drug abuse.

#### 2.2.2. Sample Collection

Subjects sat still after cleansing their faces in the laboratory of the Chinese People’s Liberation Army Air Force Specialty Medical Center (temperature (21 ± 2) °C, humidity (50 ± 10)%). The process including the following: 1. They filled out a questionnaire (basic information, living habits, etc.). 2. Half an hour later, the experimenter wore aseptic gloves and used Sebutape^®^ test strips to stick on the zygomatic bone of the left cheek of the subjects for 3 min, and then removed them, rolled them up, and placed them in an EP tube, which was stored in a refrigerator at −80 °C and prevented from oxidization. 3. Frontal images of the face were captured with a CBS cloud microscope, and then physiological indicators of the face were measured.

The physiological parameters included skin stratum corneum moisture content, TEWL, pH value, sebum, skin elasticity (R2, Q1), L/a/b value, and ITA value. Skin hydration was tested using a Corneometer CM 825, TEWL was tested using a Tewameter TM 300, sebum was tested using a sebumeter SM 815, pH value was tested using a Skin-pH-Meter PH 905, skin elasticity was tested using a Cutometer dual MPA 580, and L/a/b value and ITA value were tested using a colorimeter CL 440. The CBS Cloud Scope analyzed the skin using different polarized light, measuring indicators including pore counts, red area size, concentration of red area, brown area size, surface pigment, and deep pigment.

#### 2.2.3. Lipid Sample Processing

In this experiment, lipids were collected using Sebutape^®^ sebaceous patches. The improved Bligh and Dyer lipid extraction method was used to extract lipids, and the extraction process was as follows: 1. Lipid samples were taken out of a refrigerator at −80 °C, and 750 μL of a mixture of chloroform and methanol (2:1) was added (referred to SOI). 2. Then, an equal volume of acetone (referred to as SOII) was added, and the solution was mixed well with a vortex mixer. 3. The sample was blown dry with high-purity nitrogen, and then reconstituted at the bottom of the EP tube with a mixture of methanol and isopropanol (1:1), centrifuged for 15 min, and the supernatant was taken in an interposed tube to await assay [10]. The entire lipid extraction experiment was operated on dry ice to prevent the lipid components from volatilizing at high temperatures.

#### 2.2.4. Liquid Chromatography and Mass Spectrometry Conditions

The column was a Waters UPLC CSH C18 (2.1 mm × 100 mm, 1.7 μm), and the mobile phases were as follows: phase A (V (acetonitrile)/V (water) = 3:2), phase B (V (acetonitrile)/ V (isopropanol) = 1:9), both phase A and phase B were spiked with 0.1% formic acid and 10 mmol/L ammonium formate at a flow rate of 0.4 mL/min, and the feed volume was 5.0 μL, and the temperature in the column was 50 °C. The elution program was as follows: it began with 40% of B phase, increased linearly to 100% in the following 15 min, maintained at 100% for 3 min, and then decreased to the initial level after 2 min and held there for 2 min to complete the injection. The elution time was 22 min each time.

The mass spectrometry was performed using Waters Xevo G2-XS Q-TOF-MS for high-resolution mass detection under electrospray ionization (ESI+) mode conditions with a mass scanning range of 50~1200 *m*/*z*. Nitrogen was used as the solvent for nebulization degassing, and leucine enkephalin (LE) (*m*/*z* = 554.2771) was used as an external standard for accurate mass calibration to verify the instrumental stability. A quality control (QC) sample was injected between every 8 samples to verify the stability of the experiment.

### 2.3. Data Processing

The lipid raw data were obtained using MassLynx4.1 software, and the data were processed using Progenesis QI V2.0 and Ezinfo 3.0 software [11]. Firstly, the collected raw data were imported into QI software, peak extraction and peak alignment were performed, all the samples were divided into T1 and T2 groups, and the compound information obtained was imported into Ezinfo software; secondly, the score plots of OPLS-DA mode were combined to observe whether there were significant differences between the groups; the differential lipids were screened by the conditions of *p* < 0.05, Fold Change > 2, and VIP > 1; finally, the ROC curve AUC value > 0.9 was utilized to further screen out the key lipids related to physiological parameters and so on. Significance analysis and graphing were performed using SPSS 26.0 and Graphpad Prism 9.5.1 to obtain *p*-values, * *p* < 0.05, ** *p* < 0.01, *** *p* < 0.001, **** *p* < 0.0001.

## 3. Results

### 3.1. Results and Analysis of Research Questionnaires in T1–T2 Groups

**Figure 1:**
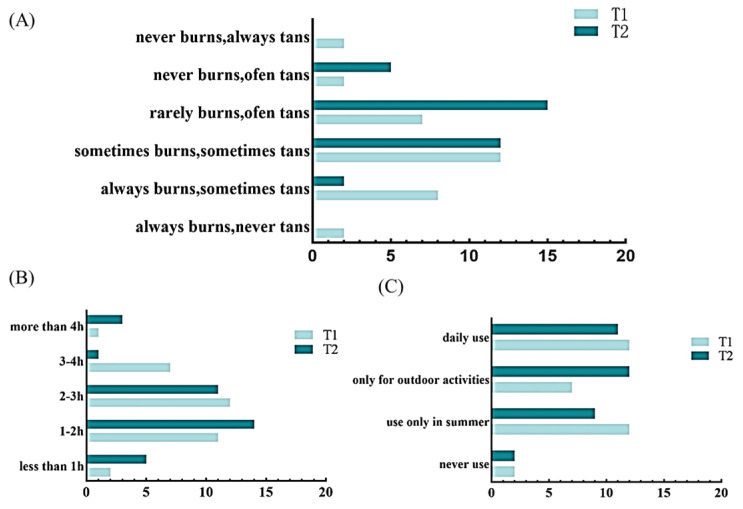
Subjects’ lifestyle habits (research questionnaires); (**A**) post-sun-exposure characteristics; (**B**) daily sun exposure hours; (**C**) sunscreen use in T1 and T2 groups.

Subjects in groups T1 and T2 were from Beijing, and the demographic measures were similar and not significantly different, in which subjects differed in the type of Fitzpatrick (cutaneous sunlight reaction typing). As shown in Figure 1, there was a significant difference (*p* = 0.03) in the post-sunburn characteristics between the T1 and T2 groups, but no significant differences in the daily hours of sun exposure or sunscreen use, where the T1 group was more susceptible to sunburn and the T2 group was more susceptible to tanning.

The Fitzpatrick skin type classification can impact skin health. The melanin content of the epidermis is also one of the main determinants of skin color, and is not linearly related to skin color [12,13]. Therefore, a quantitative study of the relationship between the physiological parameters of the skin, the facial image parameters, and the after-sun characteristics can better reflect skin characteristics [14].

### 3.2. Results and Analysis of Physiological Parameters in T1-T2 Groups

**Figure 2:**
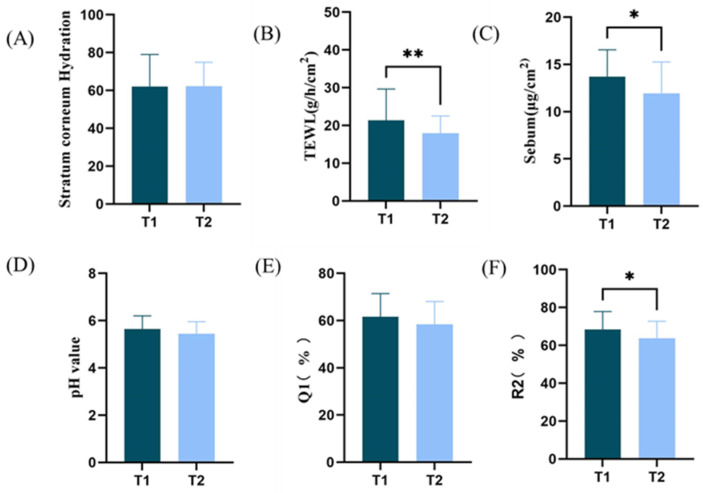
Facial physiological parameters: (**A**) skin hydration; (**B**) TWEL, (**C**) sebum; (**D**) pH; (**E**) skin elasticity Q1 value; (**F**) skin elasticity R2 value in T1 and T2 groups (* *p* < 0.05, ** *p* < 0.01).

In the T1 and T2 groups, the differences as well as significance of the indicators related to the skin barrier are shown in Figure 2, including skin hydration content (*p* = 0.9553), TWEL value (*p* = 0.0032), sebum (*p* = 0.0275), skin pH (*p* = 0.1800), Q1 value (*p* = 0.1882), and skin elasticity R2 value (*p* = 0.0485). Among them, there was a significant difference (*p* < 0.05) in about TWEL, sebum, and R2 values, all of which were greater in the T1 group than in the T2 group. Overall, the skin barrier was slightly better in group T1.

**Figure 3:**
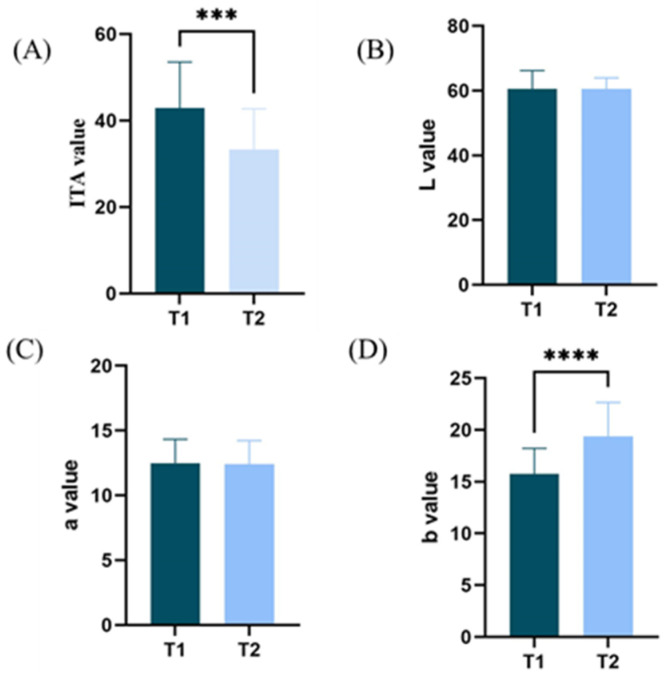
Facial physiological parameters: (**A**) ITA value; (**B**) L-value; (**C**) a-value; (**D**) b-value in T1 and T2 groups ( *** *p* < 0.001, **** *p* < 0.0001).

Skin color can reflect the integrity of the skin barrier, the sensitivity of the skin, and the skin’s response to medication, cosmetic, skin care products, and so on. Therefore, the quantitative analysis of skin color changes is of great significance [15]. Increased brightness, redness, and yellowness of facial skin are positively correlated with healthy appearance and facial attractiveness [16]. In the T1 and T2 groups, the indicators related to skin color are shown in Figure 3. The ITA value has a significant difference (*p* = 0.0002), in which the T1 group is larger than the T2 group, indicating that the T1 group has a lighter skin color; the L-value and the a-value do not have a significant difference, and the two groups of subjects are close in terms of the degree of skin fairness and redness, whereas the b value has a significant difference (*p* < 0.0001), and the skin color of the T2 group is yellower than that of the T1 group, and there is a significant difference (*p* < 0.0001). Overall, the skin color of group T1 is fairer than group T2, and this result is consistent with the results of the questionnaire research that group T1 is prone to sunburn.

**Figure 4:**
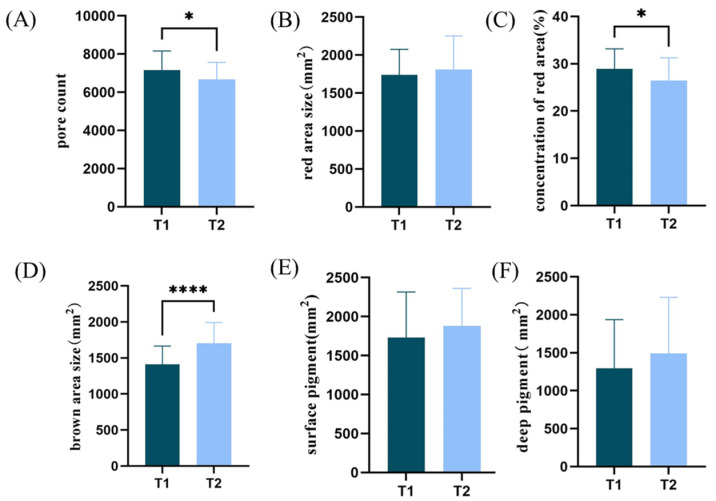
Facial physiological parameters: (**A**) pore counts; (**B**) red area size (mm^2^); (**C**) concentration of red area; (**D**) brown area size (mm^2^); (**E**) surface pigment (mm^2^); (**F**) deep pigment (mm^2^) in T1 and T2 groups (* *p* < 0.05, **** *p* < 0.0001).

In the T1 and T2 groups, the differences and significance of facial deep and superficial image indexes detected by CBS cloud microscopy are shown in Figure 4. Significant differences are observed in the number of pores (*p* = 0.0331) and red zone concentration (*p* = 0.0260); the number of pores visible and red pigment accumulation in the T1 group is greater than the T2 group; the area of the red zone (*p* = 0.5790), the area of the brown zone (*p* < 0.0001), superficial pigment RGB (*p* = 0.2582), and deep pigment Deep (*p* = 0.2470) are all greater than in the T1 group; the brown area is significantly greater in the T2 group, and there are significantly more underlying facial stains in the T2 group.

### 3.3. OPLS-DA Screening of Facial Differential Lipids in T1-T2 Groups

**Figure 5:**
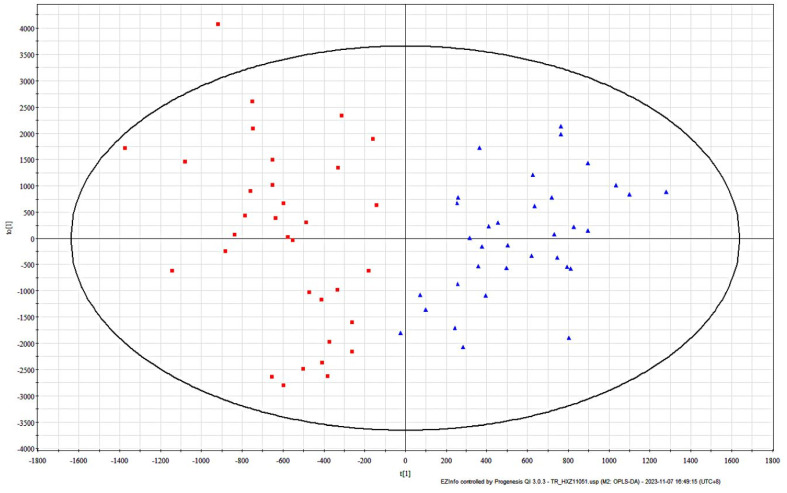
OPLS-DA score of facial skin surface lipids of subjects in T1 (red squares) and T2 (blue triangles ) groups.

After importing the mass spectrometry data of the samples into the EZinfo software, the OPLS-DA analysis mode was selected and the intergroup differences in lipid composition between the T1 and T2 groups were evaluated using score plots, as shown in Figure 5, where red squares are the T1 group, and blue triangles are the T2 group. With the segmentation line in the center, the lipid samples of female sensitive skin in group T1 are on the left side of the line, while group T2 are on the right side of the line, and the two groups are separated with no overlap, which indicates that the facial lipids of female sensitive skin are differentiated with age. Therefore, it is of great interest to study the lipid composition at the age of 36–42 years as well as at the age of 43–49 years.

### 3.4. Analysis of Facial SSL Broad Categories and Subcategory in T1-T2 Groups

**Figure 6:**
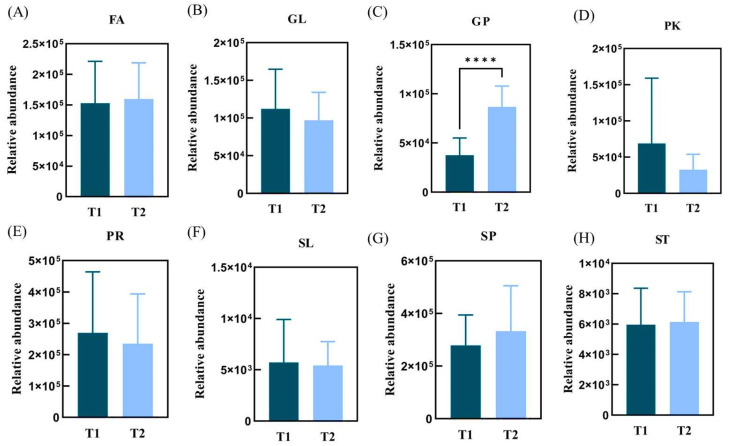
Comparison of the relative mean contents of the eight major types of lipids in T1 and T2 groups. (**A**) FA relative abundance; (**B**) GL relative abundance; (**C**) GP relative abundance; (**D**) PK relative abundance; (**E**) PR relative abundance; (**F**) SL relative abundance; (**G**) SP relative abundance; (**H**) ST relative abundance. (**** *p* < 0.0001).

Lipids are a diverse and ubiquitous group of compounds which have many key biological functions [17]. The 1078 lipids were identified in the SSL of the subjects in the T1 and T2 groups, obtained in eight major groups: GP (Glycerophospholipids), GL (Glycerolipids), FA (Fatty Acyls), SP (Sphingolipids), ST (Sterol Lipids), SL (Saccharolipids), PR (Prenol Lipids), and PK (Polyketides). Among them, there were 122 GP, 133 GL, 355 FA, 346 SP, 45 ST, only 1 SL, 36 PR, and 40 PK. The difference in the content of major lipid classes in the skin SSL of the T1 and T2 groups is shown in Figure 6: four types of lipids were higher in the T1 group, including the GL, PK, PR, and SL types; while the FA, GP, SP, and ST types were higher in the T2 group; and the GP type of lipids in the T2 group (*p* < 0.0001) was significantly higher than that in the T1 group, with the largest number of lipids in the fatty acyls class (FA), followed by sphingolipids (SP). Overall, causes of skin barrier damage in patients with SS are related to the amount and type of lipids [7]. Thus, GP lipids were predicted to be associated with the aging of sensitive skin in both age groups. GP differed significantly in the 36–42- and 43–49-year-old female sensitive skin populations, and it is very important to further analyze this group of lipids.

**Figure 7:**
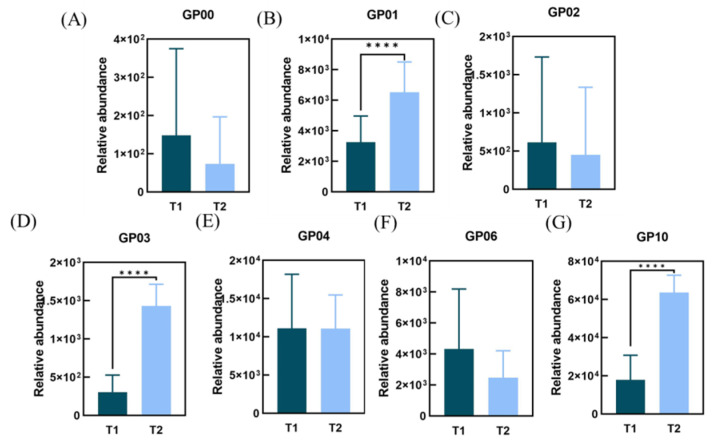
Comparison of the relative average content of facial GP subclass lipids in subjects in T1 and T2 groups. (**A**) GP00 lipids; (**B**) GP01 lipids; (**C**) GP02 lipids; (**D**) GP03 lipids; (**E**) GP04 lipids; (**F**) GP06 lipids; (**G**) GP10 lipids. (**** *p* < 0.0001).

Based on the relative trends of the eight lipid classes, subclasses of lipids with the same trends were screened. As can be seen above, the GP class (*p* < 0.0001) was significantly higher in the T2 group. Further analysis of the subclasses of GP lipids in Figure 7 shows that a total of three subclasses were significantly different (*p* < 0.05) and were consistent with the trend of change in the major classes to which they belonged. In the T2 group, the subclasses of lipids that were significantly increased included phosphatidylcholine PC (GP01, *p* < 0.0001), phosphatidylserine PS (GP03, *p* < 0.0001), and phosphatidic acid PA (GP10, *p* < 0.0001).

### 3.5. Facial VLCM Lipid Screening and ROC Prediction Analysis in T1-T2 Groups

**Figure 8:**
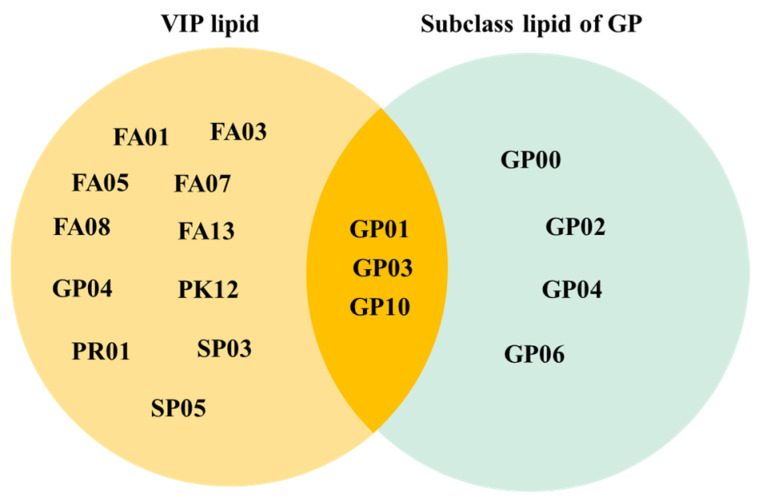
VLCM lipid screening in T1 and T2 groups.

The 1078 identified lipids were conditionally screened with *p* < 0.05, Fold Change > 2, and VIP > 1. A total of 47 differentiated lipids were obtained, and the VIP lipids with the same trend of change in the major classes and subclasses to which they belonged and with significance were selected and defined as VLCM (VIP lipids consistent with trends in category and main class) lipids. As shown in Figure 8, VIP subclass lipids included FA01, FA03, FA05, FA07, FA08, FA13, GP04, PK12, PR01, SP03, SP05, GP01, GP03, and GP10, and subclass lipids of GP included GP00, GP01, GP02, GP03, GP04, GP06, and GP10. Subclasses of lipids common to both included GP01, GP03, and GP10, and there were nine VLCM lipids belonging to these three lipid subclasses. The nine VLCM lipids included PS(O-18:0/20:5(5Z,8Z,11Z,14Z,17Z)), PS(P-16:0/12:0), PA(O-16:0/20:5(5Z,8Z,11Z,14Z,17Z)), PA(O-16:0/22:2(13Z,16Z)), PC(O-16:0/22:5(7Z,10Z,13Z,16Z,19Z)), PC(16:0/18:3(6Z,9Z,12Z)), PC(9:0/0:0), PC(19:3(10Z,13Z,16Z)/0:0), and PS(2-OMe-21:0/0:0), whose structures or properties can be found in the Lipid Maps database by Compound ID. The compound IDs of the nine VLCM lipids are LMGP03030001, LMGP03020085, LMGP10020076, LMGP10020017, LMGP01020066, LMGP01010598, LMGP01050068, LMGP01050003, and LMGP03060020, and the nine lipids were significantly different, and the SSL content of the T2 group was higher than T1, as shown in Table 1.

**Figure 9:**
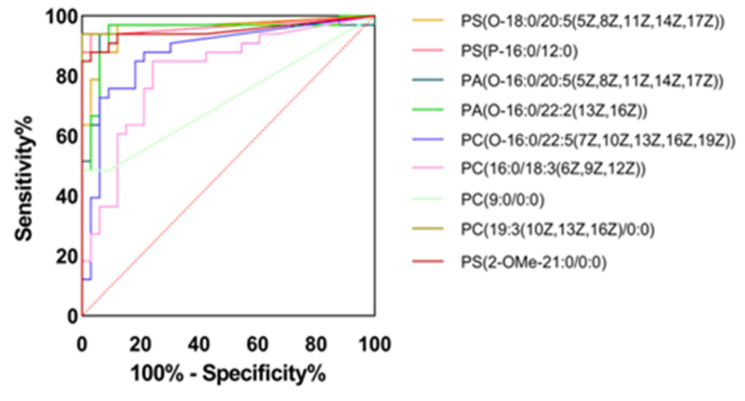
Graph of ROC analysis results of VLCM lipids in T1 and T2 groups.

The area under the receiver operating characteristic (ROC) curve (AUC) is commonly used for assessing the discriminative ability of prediction models and can also be used to assess the accuracy of biomarkers [18]. In this study, nine VLCM lipids were analyzed using the ROC curve (receiver operating characteristic), and an AUC value closer to 1 indicated a greater importance of the lipid in assessing age-related changes in sensitive skin. As shown in Figure 9, the lipids with AUC values > 0.9 of the ROC curves were PS(O-18:0/20:5(5Z,8Z,11Z,14Z,17Z)), PC(19:3(10Z,13Z,16Z)/0:0), PS(P-16:0/12:0), PA(O-16:0/22:2(13Z,16Z)), PA(O-16:0/20:5(5Z,8Z,11Z,14Z,17Z)), and PS(2-OMe-21:0/0:0) for a total of six lipids. The compound IDs of the six lipids were LMGP03020085, LMGP01050003, LMGP03030001, LMGP03030001, LMGP10020076, and LMGP10020076. With age, facial skin aging in women with sensitive skin is strongly associated with these six key lipids, which were further visualized to analyze the correlation between key lipids and physiological parameters.

### 3.6. Correlation Visual Analysis of Questionnaires, Physiological Parameters, and Key Lipids in T1–T2 Groups

#### 3.6.1. Correlation Analysis of Questionnaires and Physiological Parameters

**Figure 10:**
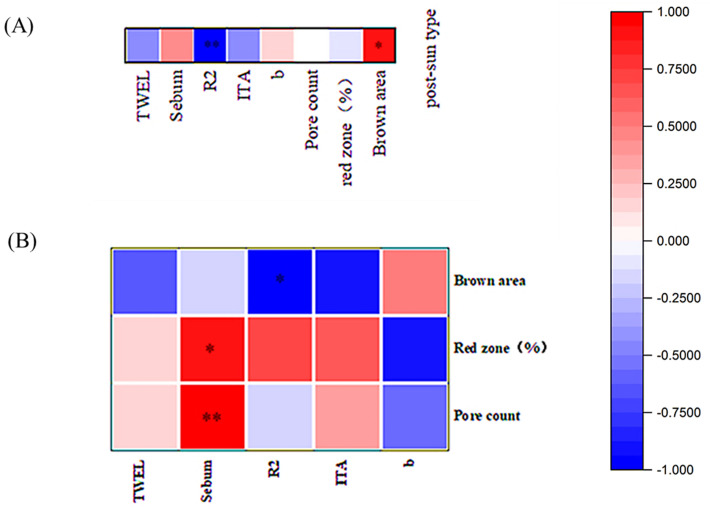
Spearman’s correlation analysis of subjects’ post-sun exposure characteristics and skin aging parameters. (**A**) correlation analysis of subjects’ post-sun exposure characteristics and physiological parameters; (**B**) correlation analysis of subjects’ physiological parameters and facial image parameters. (* *p* < 0.05, ** *p* < 0.01).

Statistically significant questionnaire findings and physiological parameters were subjected to Spearman analysis. Spearman’s rank correlation is ubiquitous in biomedical research because of its simple interpretation, robustness, and ability to capture nonlinear correlations [19]. As shown in Figure 10, post-sun characteristics had a significant negative correlation with elasticity R2, and a significant positive correlation with the brown area; the more easily a subject tanned, the less elastic the skin, and the higher the pore count; sebum were significantly correlated with the concentration of red areas andpore count.; the more oil secretion the subject had, the more pores the subject had, and the more likely they were to have redness on their face; there was a significant negative correlation between skin elasticity R2 and brown area size; the more elastic the subject’s skin, the fewer the facial stains.

Different forms of ultraviolet (UV) light, including UVA and UVB, have different effects on the skin. When the skin is irradiated for a long period of time, the activity of fibroblasts is reduced, and the activity of fibroblast-derived elastase is elevated, which leads to a decrease in the elasticity of the skin, hyperpigmentation, the decomposition of collagen fibers of types I, II, and III [20,21], and an increase in the pore count in the skin; many endogenous and exogenous factors are known to cause enlarged pores, but excessive sebum secretion has long been one of the main reasons for enlarged pores and increased pore count [22]. Recent studies have revealed that UVA, UVB, and UVC light can all cause biomechanical degradation in human stratum corneum, the outermost layer of skin, due to the dispersion of cell–cell junction proteins away from their typical intercellular locations [21]. Therefore, it is imperative to protect your skin from UV rays.

#### 3.6.2. Correlation Analysis of VLCM Lipids and Physiological Parameters

**Figure 11:**
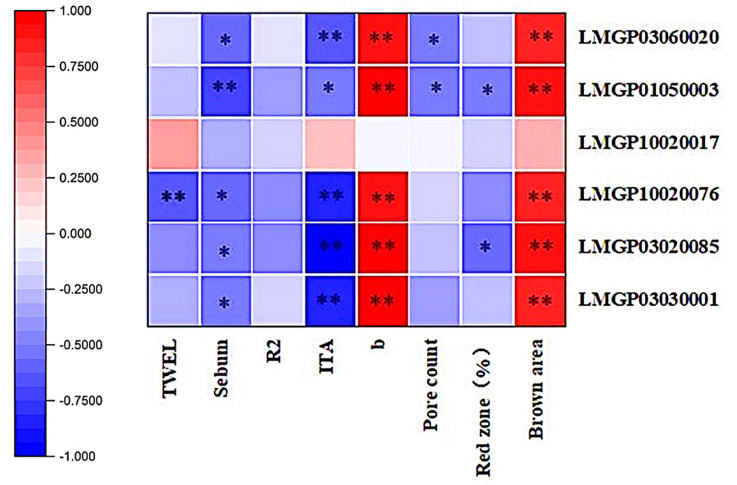
Spearman correlation analysis of key lipids and statistically significant physiological parameters. (* *p* < 0.05, ** *p* < 0.01).

Lipids of AUC values > 0.9 in the ROC curve and statistically significant physiological parameters were subjected to Spearman analysis, as shown in Figure 11. All five lipids— LMGP03060020, LMGP01050003, LMGP10020076, LMGP03020085, and LMGP03030001—correlated significantly and negatively with sebum and ITA, and significantly and positively with b-value and brown zone area; LMGP10020076 was significantly and negatively correlated with TWEL; LMGP01050003 and LMGP03020085 were significantly and negatively correlated with red area concentration; LMGP03060020 and LMGP01050003 were significantly and negatively correlated with pore count.

Overall, one GP01 lipid, three GP03 lipids, and one GP10 lipid were significantly correlated with physiological indices. Increased levels of these five lipid components— LMGP03060020, LMGP01050003, LMGP10020076, LMGP03020085, and LMGP03030001—will result in dull skin, while increased levels of LMGP01050003 lipid components will result in a decrease in red pigmentation of the skin, as well as a decrease in the number of pores.

## 4. Discussion

The aim of this study was to discuss the changes in physiological indicator characteristics and SSLs (with age) in a population of women with sensitive skin aged 36–42 and 43–49 years, as well as to investigate the relationship between key lipids and aging indicators. The results of this study showed that the parameters of physiological indicators and lipid composition changed with age in both groups. Five lipids—PS(O-18:0/20:5(5Z,8Z,11Z,14Z,17Z)), PC(19:3(10Z,13Z,16Z)/0:0), PS(P-16:0/12:0), PA(O-16. 0/20:5(5Z,8Z,11Z,14Z,17Z)), and PS(2-OMe-21:0/0:0)—significantly correlated with facial aging indicators (TWEL, sebum, ITA value, b-value, pore count, concentration of red area, and brown area size). Therefore, it is of great interest to further characterize key lipid components to develop skin care products that improve the facial physiological characteristics of those with sensitive skin in the two age groups.

The results of facial physiological parameters (aging indicators) and previous studies analyzed were basically consistent. It has been shown that there is a link between sensitive skin and epidermal barrier function, and a weak epidermal barrier increases TWEL values, and TWEL values do not increase or decrease linearly with age, but are influenced by a variety of factors [23,24]. The significantly higher TWEL values for subjects in the 36–42 age group in the present study may be related to the fact that part of the subject population in this age group is in a period of low estrogen; the effects of low estrogen on the skin are an important endogenous cause of aging skin in women [25]. The concentration of the red zone and sebum was significantly higher in the T1 group, and it has been shown that increased oil secretion exacerbates sensitization, and increased sensitization further stimulates sebum secretion [26]. Skin aging is a complex biological process, manifested by a decrease in skin elasticity, and the specificity of the aging pattern in sensitive populations may lie in inflammatory aging [27]. And there may be some subjects in the 43–49 age group who are in menopause, with estrogen deficiency resulting in decreased skin barrier function. The changes include loss of collagen, elastin, fibroblast function, and vascularity, increased matrix metalloproteinase enzymatic activity [28], and reduced skin elasticity R2. With age, in terms of skin color, the sensitive population showed a constant trend of deepening skin color and increasing skin yellowness [29]. Dermatologists have reported fewer pores in sensitive skin and a positive correlation between the number of facial pores and age [30], while pore count was significantly higher in the younger group (36–42 years old). Fair-skinned individuals were more inclined to sensitive skin [31], and total discoloration in sensitive individuals showed an increasing trend with age [29], which is consistent with the trend in the area of brown areas in this study.

A review of previous studies reveals that current studies have focused on the physiological characteristics and SSLs between sensitive and non-sensitive skin at the same age, whereas the correlation between the skin aging characteristics and lipids of sensitive skin at different ages remains unclear. Lipidomic studies of the skin have demonstrated that age, gender, ethnicity, and season of the year affect the skin lipid compositions. Likewise, alterations in lipid profiles have been linked to dermatological and systemic diseases [32]. At the present time, essential fatty acids are incorporated into the cell membranes, regenerate the damaged lipid barrier of the epidermis, and restrict water loss. The unsaturated fatty acids show prominent healing effects on skin inflammation and are used in various cosmetic products [33]. In this study, five key lipids were screened, and their relationship with facial aging parameters was investigated, providing theoretical support for developing cosmetic ingredients (lipid nanoparticles) to improve the aging of sensitive skin. PS(O-18:0/20:5(5Z,8Z,11Z,14Z,17Z), PS(P-16:0/12:0), PS(2-OMe-21:0/0:0), PA(O-16:0/20:5(5Z,8Z,11Z,14Z,17Z)), and PC (19:3 (10Z,13Z,16Z)/0:0) were predicted to be associated with skin sensitivity to aging in women in the 36–42 and 42–49 age groups. A limitation of this study is that the organismal nature and function of this liposome was not explored by cellular experiments. Skin aging is closely related to metabolism, and the two interact with each other; regulating specific lipid metabolic disorders in the skin is an important anti-aging strategy [34]. Phosphoserines (PSs) are usually located in the inner layer of the cell membrane, and PSs exhibit anti-aging activity. In the young skin, PS treatment prevented UV-induced reduction in procollagen expression and inhibited UV-induced MMP-1 expression. PS also blocked UV-induced IL-6 and COX-2 gene expression in cultured fibroblasts dose-dependently. In the aged skin, PS caused increased procollagen transcription and procollagen immunostaining in the upper dermis, and a significant decrease in MMP-1 expression at both mRNA and protein levels [35]. The three lipids—PS(O-18:0/20:5(5Z,8Z,11Z,14Z,17Z), PS(P-16:0/12:0), and PS(2-OMe-21:0/0:0)—were more abundant in the 43–49 group. It is known that redness on the skin surface is dominantly dependent on hemoglobin; both vascular development and vascular extension might contribute to the redness observed on the skin surface [36]. However, increased PS(O-18:0/20:5(5Z,8Z,11Z,14Z,17Z) content decreases facial red pigmentation and decreases deep facial inflammation. Phosphatidic acid (PA) is one of the simplest glycerophospholipids and is found in very small amounts in membranes (usually about 1%). However, phosphatidic acid plays a key role in lipid anabolism as a precursor for most glycerophospholipids and is also thought to play an important role in transmitting, amplifying, and regulating a large number of intracellular signals and cellular functions [37]. Human skin is a large organ that defends the body against stimuli that influence human health, and skin lipids are one of the most important compounds in maintaining this barrier function [38]. PA(O-16:0/20:5(5Z,8Z,11Z,14Z,17Z)) lipids were significantly and negatively correlated with the TWEL, while transepidermal water loss (TEWL) is the most widely used objective measurement for assessing the barrier function of skin in both healthy individuals and patients with skin diseases that are associated with skin barrier dysfunction [39]. Phosphatidylcholine (PC) is a critically important biomolecule found in every cell of the human body. PC is the most abundant phospholipid in eukaryotic cells and positively influences cholesterol incorporation in cell membranes [40]. In recent years, the importance of phospholipid metabolism in regulating lipid, lipoprotein, and whole-body energy metabolism has been demonstrated in numerous dietary studies and knockout animal models. One class of lipids that was altered with aging was phosphatidylcholines (PCs). More specifically, one cohort indicated that the levels of several PCs in females increased with aging [41]. Cholesterol is one of the lipid components between skin cells, forming the “brick-wall structure” of the skin with keratinocytes. It has been shown that PC lipids can be used to enhance the delivery of active ingredients to bypass the bilayer lipid structure of the skin, in order to strengthen the skin’s barrier function and slow down the aging process. However, the presence of PC(19:3(10Z,13Z,16Z)/0:0) lipids may reduce skin moisture loss and minimize pore size. Therefore, this study predicted that higher levels of this lipid would be associated with attenuated facial skin aging.

In this study, by discussing the lipid composition of physiological indicators of sensitive muscle in women in the age group of 36–42 years, three key lipids were screened for significant associations with indicators of facial aging; however, the limitation of this study is that the screened lipids were not examined at the cellular level, which will be the direction of our subsequent research.

## 5. Conclusions

In this paper, we used UPLC-Q-TOF-MS to study the difference in SSL of sensitive skin between women in the 36–42 year and 43–49 year groups, combined with questionnaires and physiological parameters, and analyzed the correlation between facial key lipids and skin aging indicators. The statistical results showed obvious differences between the two age groups of women with sensitive skin. Changes in the GP lipids were previously observed, and three key lipids were obtained by analyzing the correlation between this class of lipids and physiological parameters. Statistically, skin differences between the two age groups may be related to the three lipids. These three lipids—(PS(O-18:0/20:5(5Z,8Z,11Z,14Z,17Z), PA(O-16:0/20:5(5Z,8Z,11Z,14Z,17Z)), and PC(19:3(10Z,13Z,16Z)/0:0))—provided theoretical support for the research and development of cosmetic ingredients (lipid-based ingredients) to slow down the aging of sensitive skin.

## Figures and Tables

**Table 1:** Detailed information of facial VLCM lipids in T1 and T2 groups.

Description	Formula	Compound ID	*m*/*z*	Highest Mean
PS(P-16:0/12:0)	C34H66NO9P	LMGP03030001	686.4394	T2
PS(O-18:0/20:5(5Z,8Z,11Z,14Z,17Z))	C44H78NO9P	LMGP03020085	796.5514	T2
PA(O-16:0/20:5(5Z,8Z,11Z,14Z,17Z))	C39H69O7P	LMGP10020076	681.4844	T2
PA(O-16:0/22:2(13Z,16Z))	C41H79O7P	LMGP10020017	737.5467	T2
PC(O-16:0/22:5(7Z,10Z,13Z,16Z,19Z))	C46H84NO7P	LMGP01020066	816.5846	T2
PC(16:0/18:3(6Z,9Z,12Z))	C42H78NO8P	LMGP01010598	756.5568	T2
PC(9:0/0:0)	C17H36NO7P	LMGP01050068	398.2319	T2
PC(19:3(10Z,13Z,16Z)/0:0)	C27H50NO7P	LMGP01050003	554.3197	T2
PS(2-OMe-21:0/0:0)	C28H58NO9P	LMGP03060020	606.374	T2

## Data Availability

The data presented in this study are available on request from the corresponding author. The data are not publicly available due to the test data involves working with an external company, it is necessary to communicate with the corresponding author.
